# Crystal structure and Hirshfeld surface analysis of dimethyl 4-hy­droxy-5,4′-dimethyl-2′-(toluene-4-sulfonyl­amino)­biphenyl-2,3-di­carboxyl­ate

**DOI:** 10.1107/S205698902301071X

**Published:** 2024-01-01

**Authors:** Narmina A. Guliyeva, Gleb M. Burkin, Selbi Annadurdyyeva, Victor N. Khrustalev, Zeliha Atioğlu, Mehmet Akkurt, Ajaya Bhattarai

**Affiliations:** a Department of Organic Substances and Technology of High-Molecular Compounds, SRI Geotechnological Problems of Oil, Gas and Chemistry, Azerbaijan State Oil and Industry University, Azadlig ave. 20, Az-1010 Baku, Azerbaijan; b RUDN University, 6 Miklukho-Maklaya St., Moscow 117198, Russian Federation; c Zelinsky Institute of Organic Chemistry of RAS, 4, 7 Leninsky Prospect, 119991 Moscow, Russian Federation; d Department of Aircraft Electrics and Electronics, School of Applied Sciences, Cappadocia University, Mustafapaşa, 50420 Ürgüp, Nevşehir, Türkiye; e Department of Physics, Faculty of Sciences, Erciyes University, 38039 Kayseri, Türkiye; f Department of Chemistry, M.M.A.M.C (Tribhuvan University) Biratnagar, Nepal; Texas A & M University, USA

**Keywords:** crystal structure, hydrogen bonds, Hirshfeld surface analysis, [4 + 2] cyclo­addition, furan, aryl­sulfonamides

## Abstract

In the title compound, C_25_H_25_NO_7_S, the mol­ecular conformation is stabilized by intra­molecular O—H⋯O and N—H⋯O hydrogen bonds, which form *S*(6) and *S*(8) ring motifs, respectively. The mol­ecules are bent at the S atom with a C—SO_2_—NH—C torsion angle of −70.86 (11)°. In the crystal, mol­ecules are linked by C—H⋯O and N—H⋯O hydrogen bonds, forming mol­ecular layers parallel to the (100) plane. C—H⋯π inter­actions are observed between these layers.

## Abstract 2

In the crystal, mol­ecules are connected by C—H⋯O and N—H⋯O hydrogen bonds, forming mol­ecular layers parallel to the (100) plane. These layers are connected to each other by C—H⋯π inter­actions.

## 1. Chemical context

**Figure 1:**
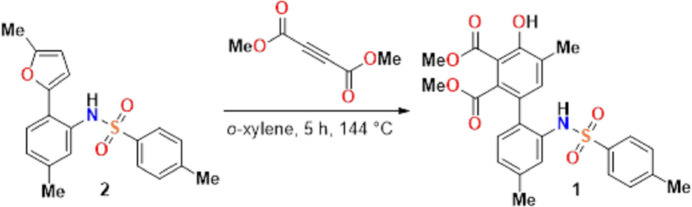
Synthesis of 4-hy­droxy-5,4′-dimethyl-2′-(toluene-4-sulfonyl­amino)­biphenyl-2,3-di­carb­oxy­lic acid dimethyl ester (**1**).

Furan contains a system of conjugated *s*-*cis*-double bonds, closed through an oxygen atom, and as a result, this heterocycle easily participates in Diels–Alder reactions. The [4 + 2] cyclo­addition of furan with acetyl­enedi­carb­oxy­lic acid esters (as alkynes) was performed for the first time to find a simple route for the preparation of *Cantharidin* (Diels & Alder, 1931 ▸). Furan reacts with esters of acetyl­enedi­carb­oxy­lic acid when heated to 373 K. The 7-oxabi­cyclo­[2.2.1]heptene scaffold, the product of the reaction between furans and alkynes, has great synthetic potential as a useful tool for the design of a broad diversity of substances with various practical properties. These cyclo­adducts have been used to construct polycyclic aromatic hydro­carbons (Eda *et al.*, 2015 ▸; Criado *et al.*, 2013 ▸). The annulated 7-oxabi­cyclo­[2.2.1]heptane moiety also acts as a framework for synthesis of mol­ecular tweezers (Murphy *et al.*, 2016 ▸; Warrener *et al.*, 1999 ▸), high-mol­ecular-weight materials (Margetić *et al.*, 2010 ▸; Vogel *et al.*, 1999 ▸) and various supra­molecular systems (Abdelhamid *et al.*, 2011 ▸; Akbari Afkhami *et al.*, 2017 ▸; Khalilov *et al.*, 2021 ▸; Safarova *et al.*, 2019 ▸). Under acid catalysis, cyclo­addition inter­mediates can be converted into phenols, cyclo­hexenoles, or substituted aromatic hydro­carbons (Zaytsev *et al.*, 2019 ▸; Zubkov *et al.*, 2012*a* ▸,*b* ▸). In this work, we continued our investigations of the cyclo­addition of dimethyl acetyl­enedi­carboxyl­ate (DMAD) with substituted furans (Zubkov *et al.*, 2009 ▸; Borisova *et al.*, 2018*a* ▸,*b* ▸). In particular, in the course of the thermic [4 + 2] cyclo­addition between DMAD and sulfamide **2**, an inter­esting sequence of reaction steps was observed: a cleavage of the ep­oxy bridge and a sigmatropic shift of the methyl group (Fig. 1 ▸). On the other hand, the biological and catalytic activity as well as coordination ability of the new sulfamide derivative **1** can be dictated by the non-covalent bond-donor or acceptor character of the substituents (Gurbanov *et al.*, 2022*a* ▸,*b* ▸; Kopylovich *et al.*, 2011*a* ▸,*b* ▸; Mahmoudi *et al.*, 2017*a* ▸,*b* ▸; Mahmudov *et al.*, 2013 ▸). 
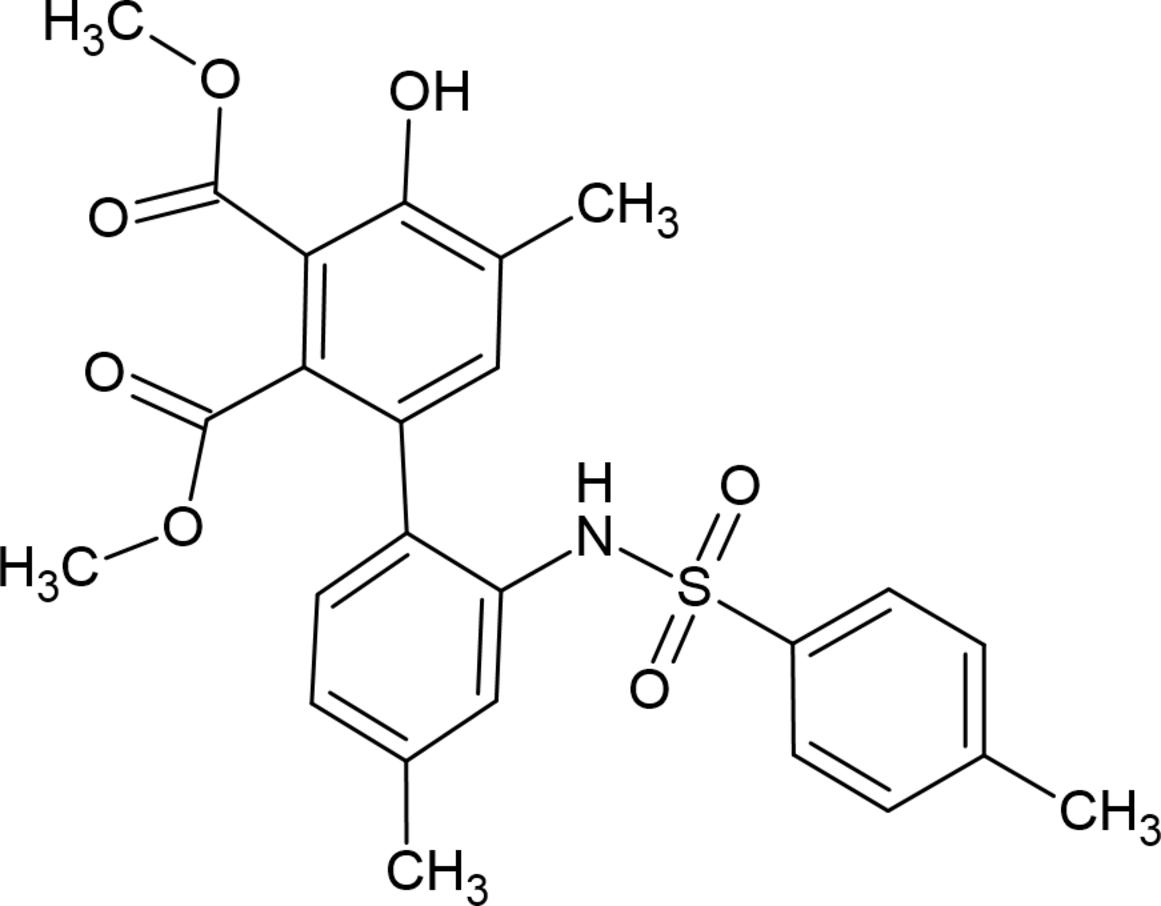


## 2. Structural commentary

**Figure 2:**
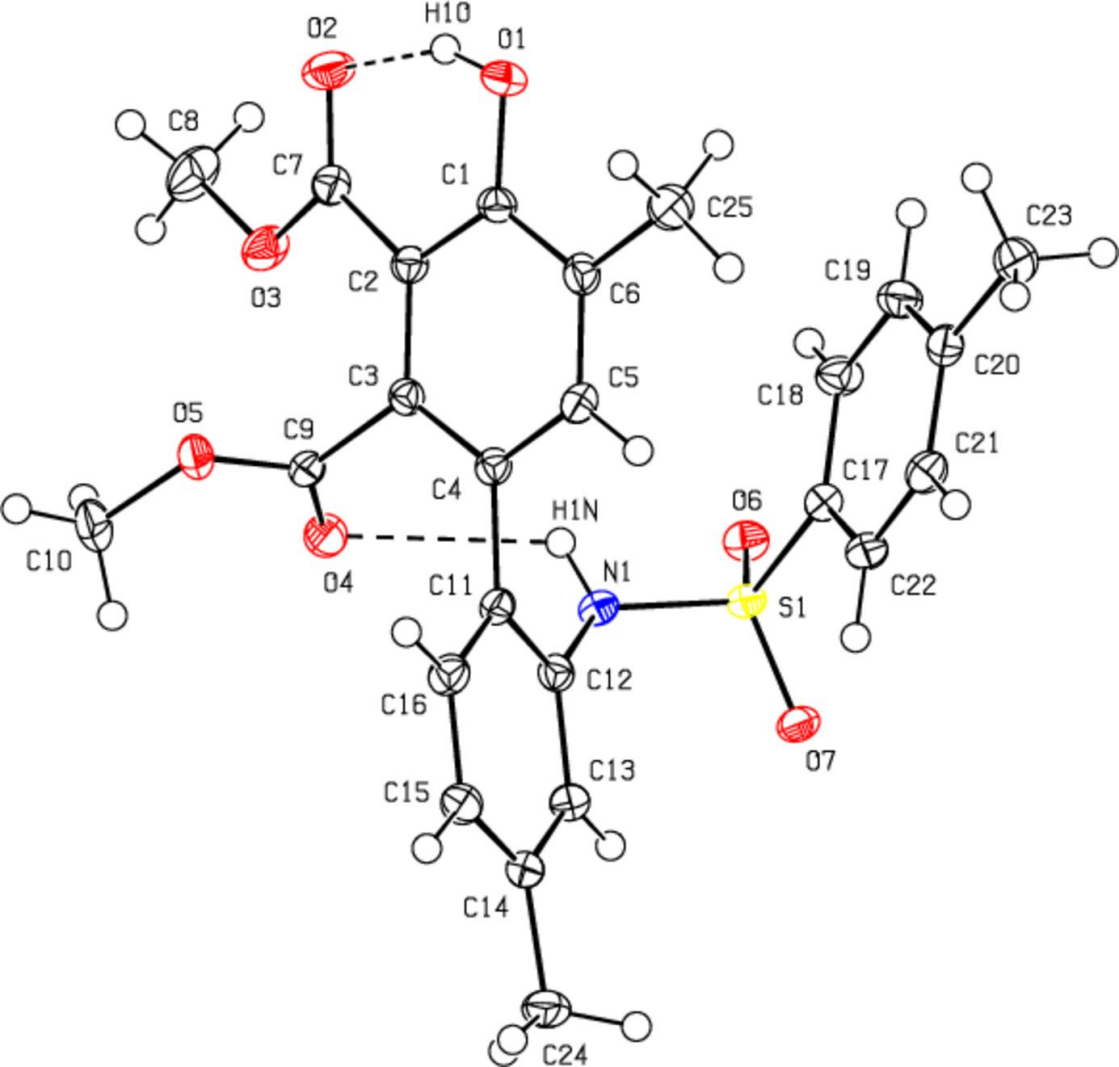
Structure and atomic numbering scheme of the title compound, shown as 50% probability ellipsoids.

In the title compound (Fig. 2 ▸), the mol­ecular conformation is stabilized by intra­molecular O—H⋯O and N—H⋯O hydrogen bonds, which form *S*(6) and *S*(8) ring motifs, respectively. Mol­ecules of the title compound are bent at the S atom with a C17—S1—N1—C12 torsion angle of −70.86 (11)°. The benzene ring (C11–C16) attached to the N atom makes a dihedral angle of 77.99 (6)° with the benzene ring (C1–C6) having the OH group, and these rings make angles of 26.98 (6) and 57.58 (6)°, respectively, with the benzene ring (C17–C22) attached to the S atom. The geometric parameters of the title compound are normal and comparable to those of the related compound listed in the *Database survey* section.

## 3. Supra­molecular features and Hirshfeld surface analysis

**Figure 3:**
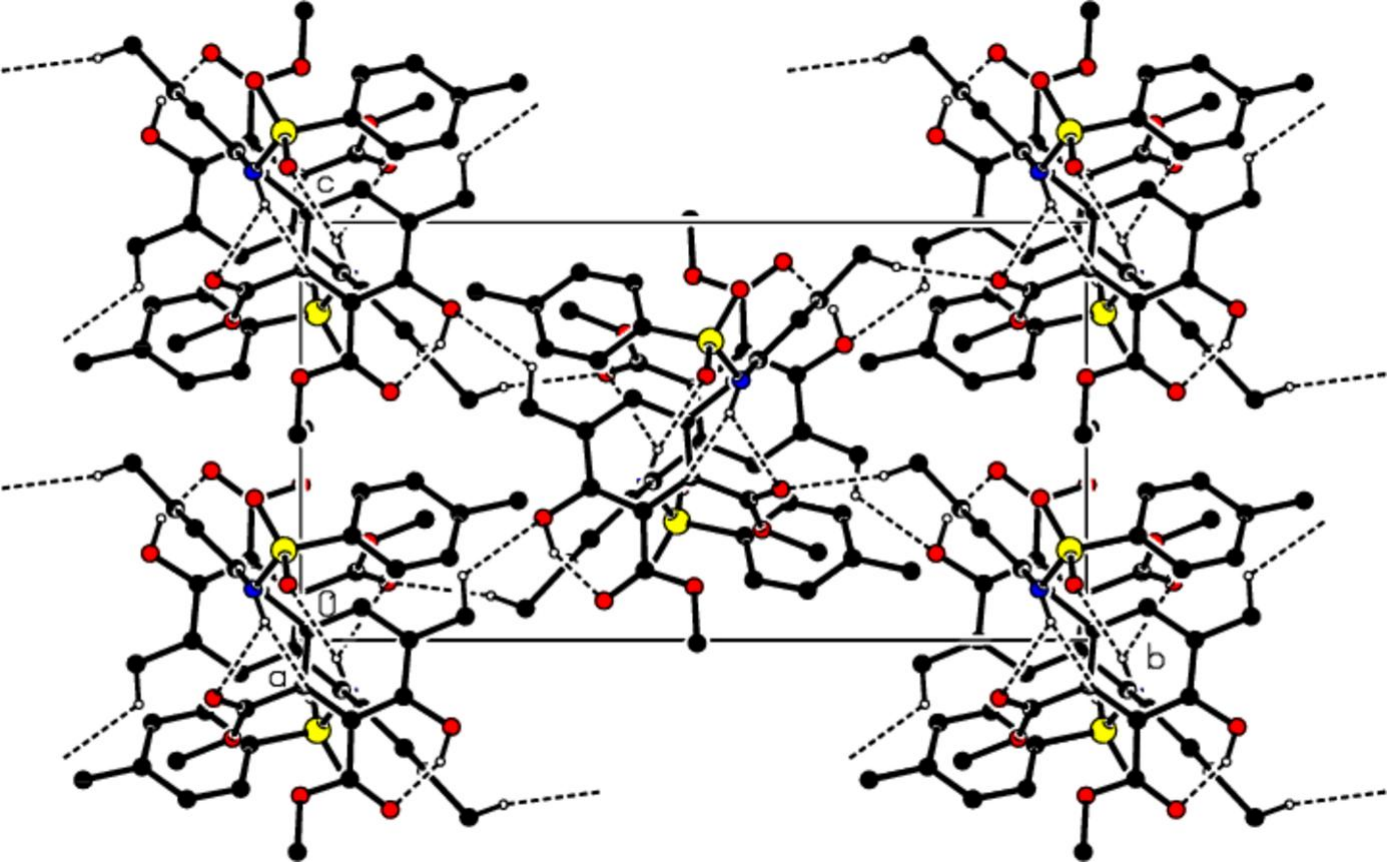
Packing of mol­ecules in the title compound with the N—H⋯O, O–H⋯O and C—H⋯O hydrogen bonds, viewed along the *a* axis.

In the crystal, mol­ecules are linked by C—H⋯O and N—H⋯O hydrogen bonds, forming mol­ecular layers parallel to the (100) plane (Table 1 ▸, Figs. 3 ▸, 4 ▸ and 5 ▸). C—H⋯π inter­actions (Table 1 ▸) between these layers also add to the crystal cohesion.

**Figure 6:**
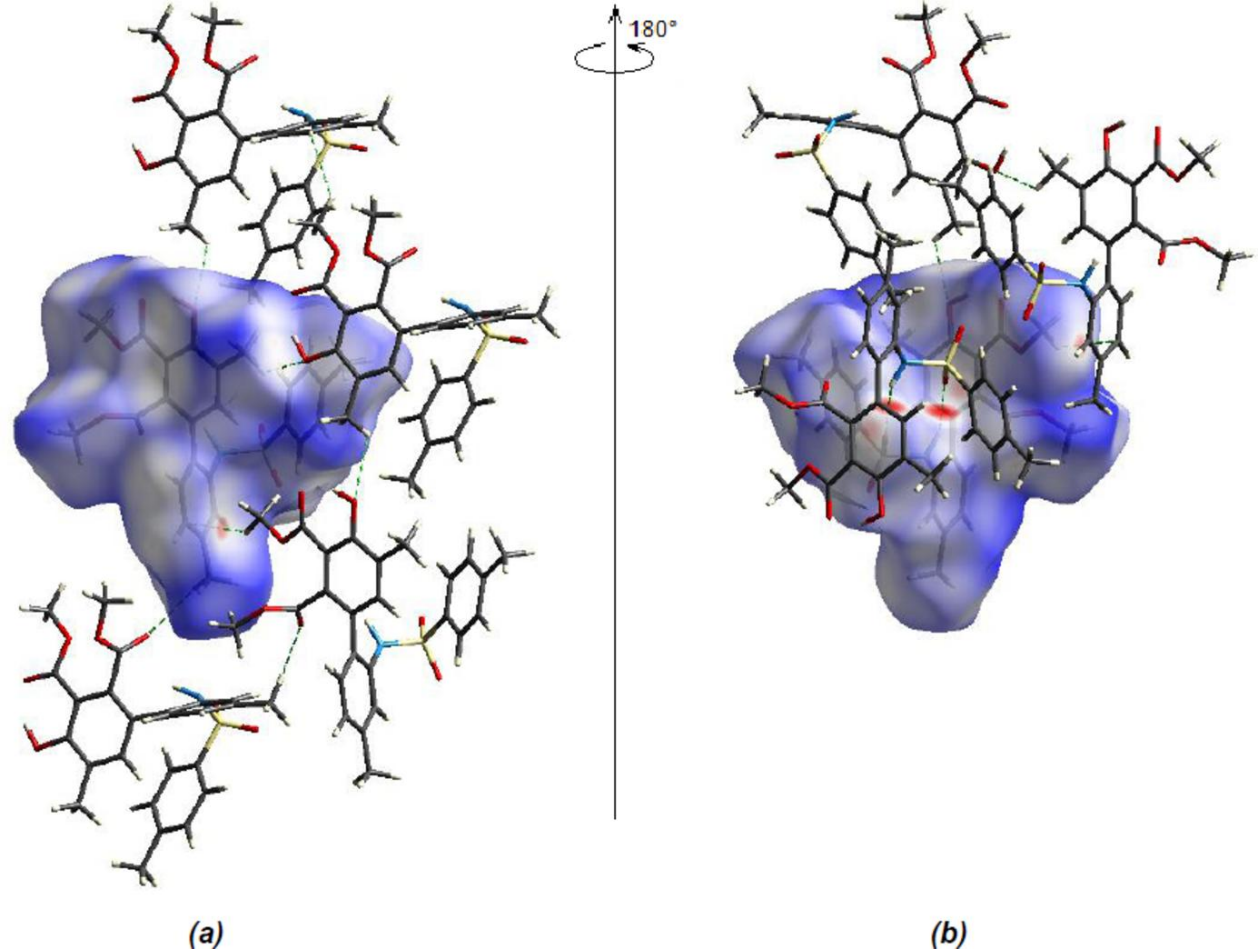
(*a*) Front and (*b*) back views of the three-dimensional Hirshfeld surface for the title compound.

To qu­antify the inter­molecular inter­actions, a Hirshfeld surface analysis was performed and *CrystalExplorer17.5* (Spackman *et al.*, 2021 ▸) was used to generate the accompanying two-dimensional fingerprint plots. Fig. 6 ▸ shows the Hirshfeld surface mapped over *d*_norm_. On the Hirshfeld surface, shorter and longer contacts are indicated by red and blue spots, respectively, and contacts with lengths about equal to the sum of the van der Waals radii are indicated by white spots. The C—H⋯O and N—H⋯O inter­actions (Tables 1 ▸ and 2 ▸) are represented by the two most significant red spots on the *d*_norm_ surface.

**Figure 7:**
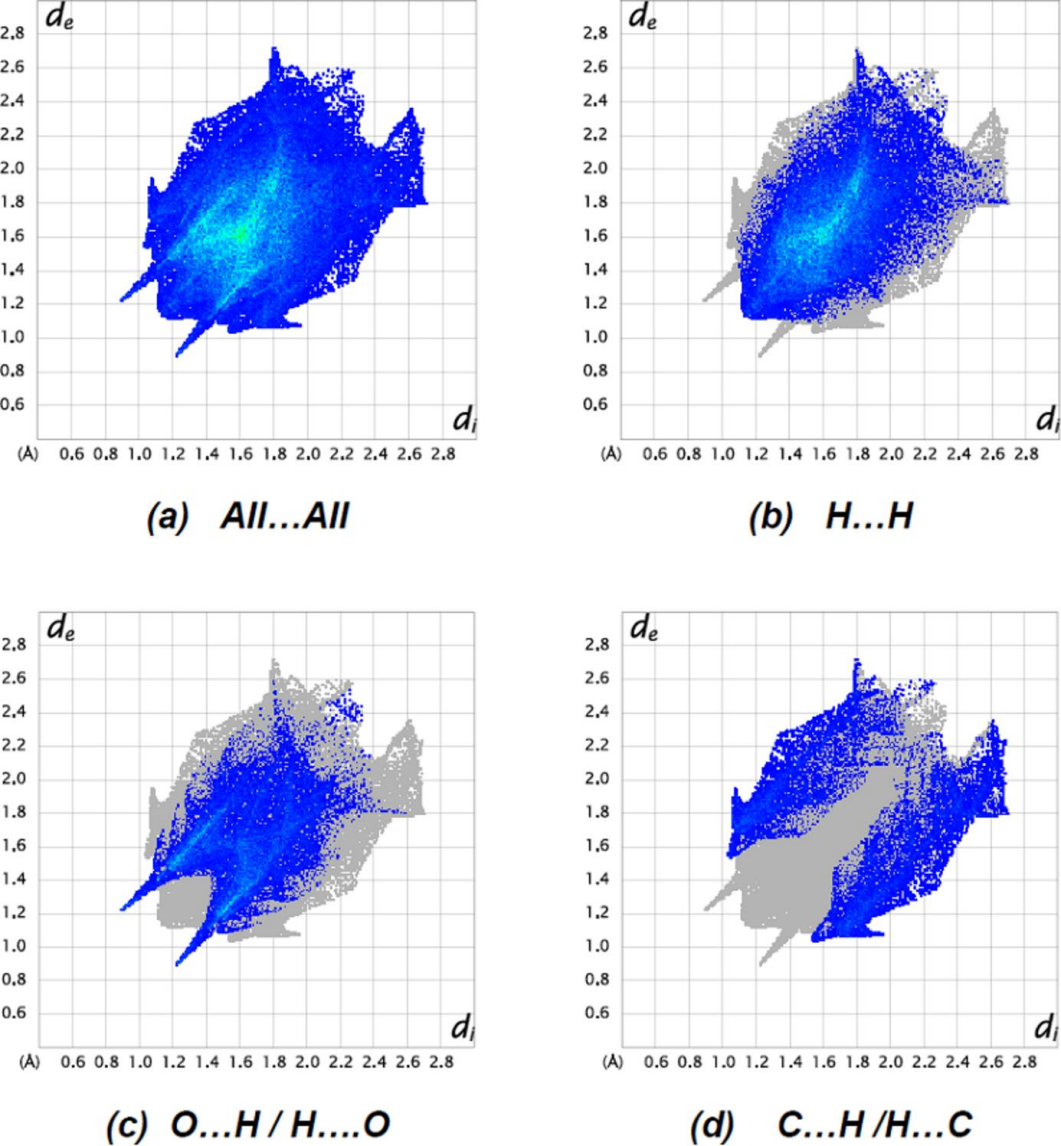
The two-dimensional fingerprint plots for the title compound showing (*a*) all inter­actions, and delineated into (*b*) H⋯H, (*c*) F⋯H/H⋯F, (*d*) O⋯H/H⋯O and (*e*) C⋯H/H⋯C inter­actions. The *d*_i_ and *d*_e_ values are the closest inter­nal and external distances (in Å) from given points on the Hirshfeld surface.

Fig. 7 ▸ depicts the two-dimensional fingerprint plots of (*d*_i_, *d*_e_) points from all the contacts contributing to the Hirshfeld surface analysis in normal mode for all atoms. The most important inter­molecular inter­actions are H⋯H contacts, contributing 52.3% to the overall crystal packing. Other inter­actions and their respective contributions are O⋯H/H⋯O (27.0%), C⋯H/H⋯C (15.2%), O⋯C/C⋯O (2.5%), O⋯O (2.0%) and N⋯H/H⋯N (1.1%). The Hirshfeld surface analysis confirms the significance of H-atom inter­actions in the packing formation. The significant frequency of H⋯H and O⋯H/H⋯O inter­actions implies that van der Waals inter­actions and hydrogen bonding are important in crystal packing (Hathwar *et al.*, 2015 ▸).

## 4. Database survey

A search of the Cambridge Structural Database (CSD, Version 5.43, last update November 2022; Groom *et al.*, 2016 ▸) for the *N*,4-di­methyl­benzene-1-sulfonamide unit, resulted in two hits, CSD refcodes EVOJAB (Shakuntala, *et al.*, 2011*a* ▸) and EVOFAX (Shakuntala, *et al.*, 2011*b* ▸).

The mol­ecule of EVOJAB (Shakuntala, *et al.*, 2011*a* ▸) is twisted about the N—S bond with a C—SO_2_—NH—C torsion angle of 44.55 (17)°. The two aromatic rings are inclined to each other by 66.2 (1)°. In the crystal, N—H⋯O hydrogen bonds link the mol­ecules into infinite chains parallel to the *b* axis. Mol­ecules of EVOFAX (Shakuntala, *et al.*, 2011*b* ▸), are bent at the S atom with a C—SO_2_—NH—C torsion angle of 57.7 (2)°. The benzene rings are rotated relative to each other by 68.1 (1)°. In the crystal, N—H⋯O(S) hydrogen bonds pack the mol­ecules into infinite chains parallel to the *b* axis.

## 5. Synthesis and crystallization

Dimethyl but-2-ynedioate (87.6 µL, 0.7 mmol) was added to a solution of 4-methyl-*N*-(5-methyl-2-(5-methyl­furan-2-yl)phen­yl)benzene­sulfonamide (100 mg, 0.3 mmol) in *o*-xylene (5 mL). The mixture was refluxed for 5 h. After cooling of the reaction to r.t, the solvent was evaporated under reduced pressure and the crude product was purified by column chromatography (eluent: from hexane to ethyl acetate). The title compound was obtained as a colourless powder, yield 68%, 97 mg (0.2 mmol); m.p. > 523 K (with decomp.). A single crystal of the title compound was grown from a mixture of hexane and ethyl acetate. IR (KBr), *ν* (cm^−1^): *br*. 3277 (NH, OH), 1745, 1671 (CO_2_), 1353 (ν_as_ SO_2_), 1238 (C—OH), 1172 (ν_s_ SO_2_). ^1^H NMR (700.2 MHz, CDCl_3_) (*J*, Hz): *δ* 11.31 (*s*, 1H, OH), 7.52 (*s*, 1H, NH), 7.42 (*d*, *J* = 8.2, 2H, H Ar), 7.18 (*d*, *J* = 8.2, 2H, H Ar), 6.96 (*d*, *J* = 7.6, 1H, H Ar), 6.85 (*d*, *J* = 7.6, 1H, H Ar), 6.71 (*s*, 1H, H Ar), 6.08 (*s*, 1H, H Ar), 3.92 (*s*, 3H, OCH_3_), 3.51 (*s*, 3H, OCH_3_), 2.43 (*s*, 3H, CH_3_), 2.39 (*s*, 3H, CH_3_), 2.06 (*s*, 3H, CH_3_). ^13^C{^1^H} NMR (176.1 MHz, CDCl_3_): *δ* 169.5, 168.7, 159.9, 143.3, 139.4, 137.3, 136.9, 134.1, 133.0, 130.7, 129.5 (2C), 129.4, 128.5, 127.1 (2C), 126.3, 125.7, 124.7, 107.9, 53.1, 52.3, 21.6, 21.4, 16.0. MS (ESI) *m/z*: [*M* + H]^+^ 484. Elemental analysis calculated (%) for C_25_H_25_NO_7_S: C 62.10, H 5.21, N 2.90, S 6.63; found: C 61.87, H 5.48, N 3.09, S 6.37.

## 6. Refinement

Crystal data, data collection and structure refinement details are summarized in Table 3 ▸. C-bound H atoms were included in the refinement using the riding-model approximation with C—H distances of 0.95–0.98 Å, and with *U*_iso_(H) = 1.2 or 1.5*U*_eq_(C). The H atoms of the NH and OH groups were found in a difference map and refined freely [N1—H1*N* = 0.836 (18) Å and O1—H1*O* = 0.91 (2) Å].

## Supplementary Material

Crystal structure: contains datablock(s) I. DOI: 10.1107/S205698902301071X/jy2041sup1.cif

Structure factors: contains datablock(s) I. DOI: 10.1107/S205698902301071X/jy2041Isup2.hkl

Supporting information file. DOI: 10.1107/S205698902301071X/jy2041Isup3.cml

CCDC reference: 2314397

Additional supporting information:  crystallographic information; 3D view; checkCIF report

## Figures and Tables

**Figure 4:**
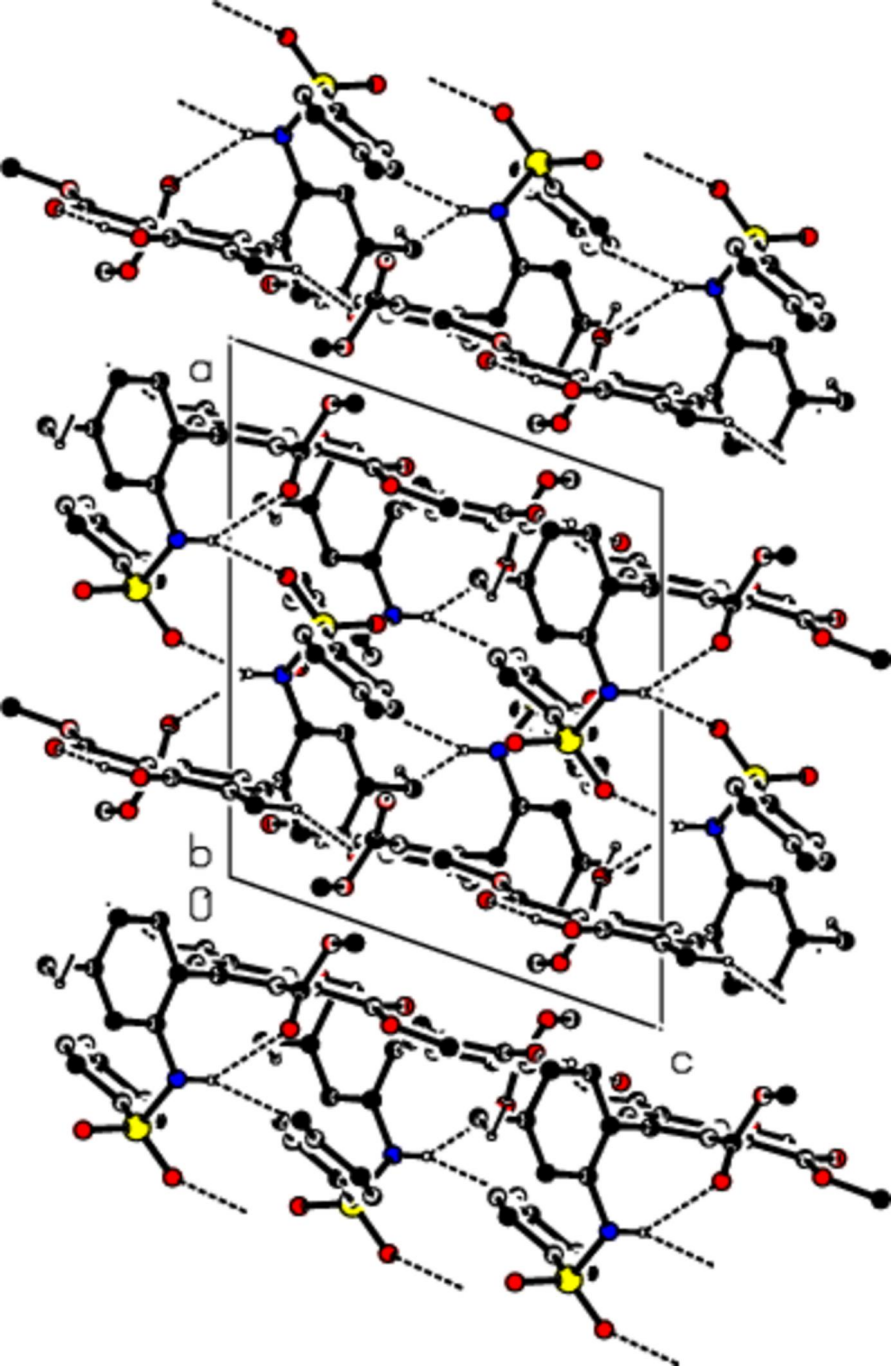
Packing of mol­ecules in the title compound, viewed along the *b* axis.

**Figure 5:**
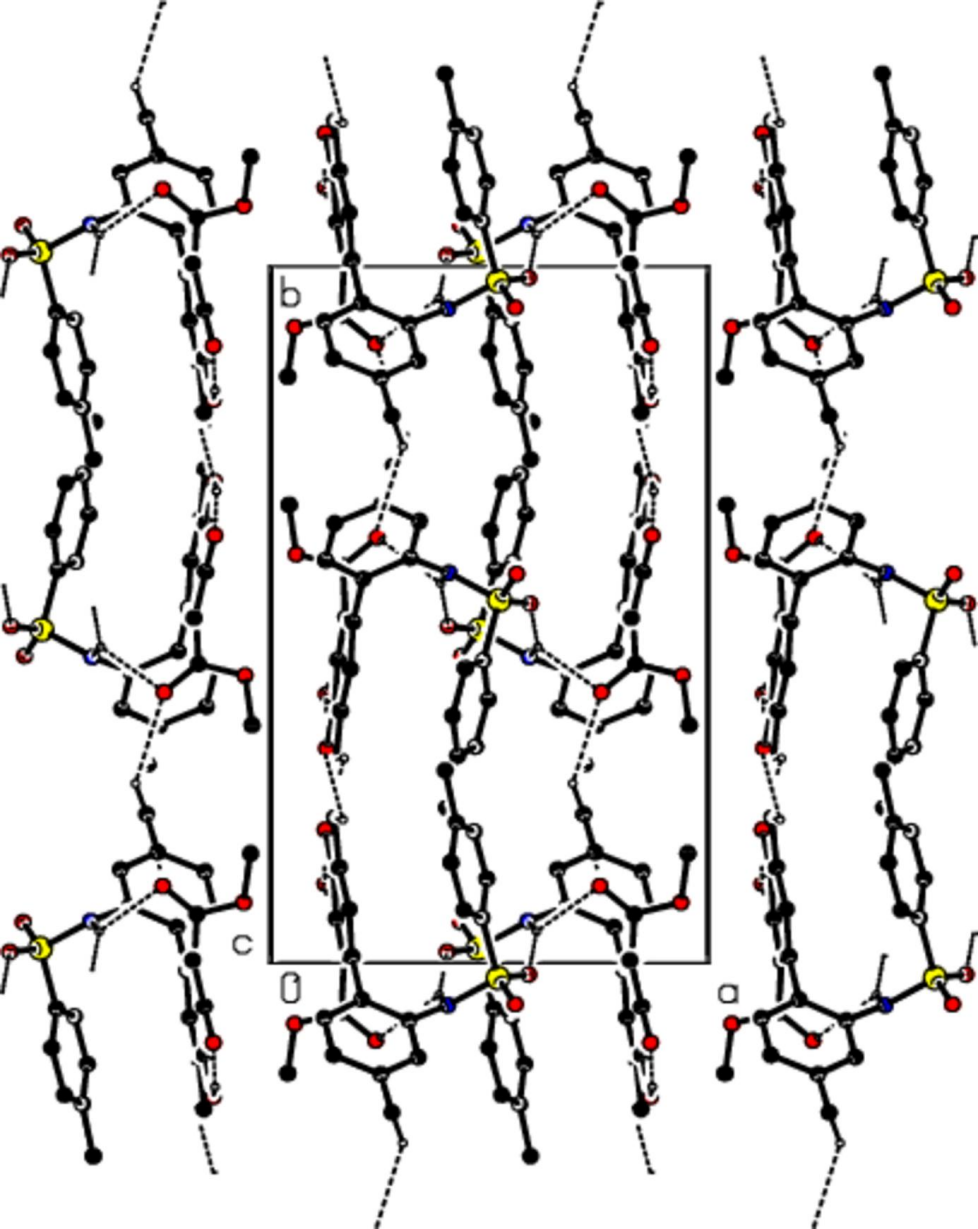
Packing of mol­ecules in the title compound, viewed along the *c* axis.

**Table 1:** Hydrogen-bond geometry (Å, °) *Cg*1 is the centroid of the C1–C6 ring.

*D*—H⋯*A*	*D*—H	H⋯*A*	*D*⋯*A*	*D*—H⋯*A*
N1—H1*N*⋯O4	0.836 (18)	2.507 (18)	3.0185 (14)	120.5 (15)
N1—H1*N*⋯O6^i^	0.836 (18)	2.280 (18)	3.0643 (14)	156.4 (18)
O1—H1*O*⋯O2	0.91 (2)	1.72 (2)	2.5596 (14)	152 (2)
C24—H24*A*⋯O4^ii^	0.98	2.60	3.3784 (16)	137
C25—H25*C*⋯O1^iii^	0.98	2.59	3.2177 (17)	122
C16—H16⋯*Cg*1^iv^	0.95	2.61	3.4780 (14)	153

Symmetry codes: (i) 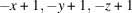 ; (ii) 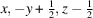 ; (iii) 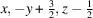 ; (iv) 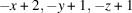 .

**Table 2:** Summary of short inter­atomic contacts (Å) in the title compound

Contact	Distance	Symmetry operation
H15⋯O1	2.67	2 − *x*, 1 − *y*, 1 − *z*
H1*O*⋯H25*C*	2.40	*x*, 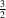 − *y*, 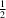 + *z*
H10*C*⋯O2	2.71	2 − *x*, 1 − *y*, 2 − *z*
H1*N*⋯O6	2.28	1 − *x*, 1 − *y*, 1 − *z*
O4⋯H24*A*	2.60	*x*, 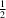 − *y*, 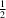 + *z*
H22⋯H22	2.47	1 − *x*, 1 − *y*, −*z*
H8*C*⋯C15	2.65	*x*, *y*, 1 + *z*
H13⋯H19	2.57	1 − *x*, − 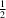 + *y*, 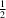 − *z*

**Table 3:** Experimental details

Crystal data	
Chemical formula	C_25_H_25_NO_7_S
*M*_r_	483.52
Crystal system, space group	Monoclinic, *P*2_1_/*c*
Temperature (K)	100
*a*, *b*, *c* (Å)	12.52978 (9), 18.87277 (12), 10.63916 (7)
β (°)	109.4092 (8)
*V* (Å^3^)	2372.88 (3)
*Z*	4
Radiation type	Cu *K*α
μ (mm^−1^)	1.61
Crystal size (mm)	0.22 × 0.20 × 0.15

Data collection	
Diffractometer	XtaLAB Synergy, Dualflex, HyPix
Absorption correction	Multi-scan (*CrysAlis PRO*; Rigaku OD, 2021 ▸)
*T*_min_, *T*_max_	0.654, 1.000
No. of measured, independent and observed [*I* > 2σ(*I*)] reflections	37027, 5051, 4842
*R*_int_	0.044
(sin θ/λ)_max_ (Å^−1^)	0.634

Refinement	
*R*[*F* ^2^ > 2σ(*F* ^2^)], *wR*(*F* ^2^), *S*	0.033, 0.088, 1.03
No. of reflections	5051
No. of parameters	321
H-atom treatment	H atoms treated by a mixture of independent and constrained refinement
Δρ_max_, Δρ_min_ (e Å^−3^)	0.33, −0.44

Computer programs: *CrysAlis PRO* (Rigaku OD, 2021 ▸), *SHELXT2016/6* (Sheldrick, 2015*a* ▸), *SHELXL2016/6* (Sheldrick, 2015*b* ▸), *ORTEP-3 for Windows* (Farrugia, 2012 ▸) and *PLATON* (Spek, 2020 ▸).

## supplementary crystallographic information

### Crystal data

**Table:**

C_25_H_25_NO_7_S	*F*(000) = 1016
*M**_r_* = 483.52	*D*_x_ = 1.354 Mg m^−^^3^
Monoclinic, *P*2_1_/*c*	Cu *K*α radiation, λ = 1.54184 Å
*a* = 12.52978 (9) Å	Cell parameters from 26913 reflections
*b* = 18.87277 (12) Å	θ = 4.4–77.8°
*c* = 10.63916 (7) Å	µ = 1.61 mm^−^^1^
β = 109.4092 (8)°	*T* = 100 K
*V* = 2372.88 (3) Å^3^	Prism, colourless
*Z* = 4	0.22 × 0.20 × 0.15 mm

### Data collection

**Table:**

XtaLAB Synergy, Dualflex, HyPix diffractometer	4842 reflections with *I* > 2σ(*I*)
Radiation source: micro-focus sealed X-ray tube	*R*_int_ = 0.044
φ and ω scans	θ_max_ = 77.8°, θ_min_ = 3.7°
Absorption correction: multi-scan (CrysAlisPro; Rigaku OD, 2021)	*h* = −15→15
*T*_min_ = 0.654, *T*_max_ = 1.000	*k* = −22→23
37027 measured reflections	*l* = −13→13
5051 independent reflections	

### Refinement

**Table:**

Refinement on *F*^2^	Secondary atom site location: difference Fourier map
Least-squares matrix: full	Hydrogen site location: mixed
*R*[*F*^2^ > 2σ(*F*^2^)] = 0.033	H atoms treated by a mixture of independent and constrained refinement
*wR*(*F*^2^) = 0.088	*w* = 1/[σ^2^(*F*_o_^2^) + (0.0419*P*)^2^ + 1.207*P*] where *P* = (*F*_o_^2^ + 2*F*_c_^2^)/3
*S* = 1.03	(Δ/σ)_max_ = 0.001
5051 reflections	Δρ_max_ = 0.33 e Å^−^^3^
321 parameters	Δρ_min_ = −0.44 e Å^−^^3^
0 restraints	Extinction correction: SHELXL, Fc^*^=kFc[1+0.001xFc^2^λ^3^/sin(2θ)]^-1/4^
Primary atom site location: difference Fourier map	Extinction coefficient: 0.00097 (9)

### Special details

**Table:**

Experimental. CrysAlisPro 1.171.41.117a (Rigaku OD, 2021) Empirical absorption correction using spherical harmonics, implemented in SCALE3 ABSPACK scaling algorithm.
Geometry. All esds (except the esd in the dihedral angle between two l.s. planes) are estimated using the full covariance matrix. The cell esds are taken into account individually in the estimation of esds in distances, angles and torsion angles; correlations between esds in cell parameters are only used when they are defined by crystal symmetry. An approximate (isotropic) treatment of cell esds is used for estimating esds involving l.s. planes.

### Fractional atomic coordinates and isotropic or equivalent isotropic displacement parameters (Å^2^)

**Table:**

	*x*	*y*	*z*	*U*_iso_*/*U*_eq_	
S1	0.47540 (2)	0.47876 (2)	0.28445 (3)	0.01572 (9)	
O1	0.87334 (8)	0.69147 (5)	0.70753 (10)	0.0219 (2)	
H1O	0.8780 (19)	0.6776 (12)	0.791 (2)	0.054 (6)*	
O2	0.87491 (9)	0.61396 (5)	0.90556 (9)	0.0289 (2)	
O3	0.83061 (9)	0.49945 (5)	0.87207 (9)	0.0254 (2)	
O4	0.75736 (8)	0.38919 (5)	0.63886 (9)	0.0229 (2)	
O5	0.94306 (8)	0.41300 (5)	0.73522 (9)	0.0245 (2)	
O6	0.40571 (7)	0.48386 (5)	0.36703 (9)	0.01983 (19)	
O7	0.43510 (7)	0.44113 (5)	0.16051 (8)	0.01987 (19)	
N1	0.59121 (9)	0.43960 (5)	0.37778 (10)	0.0167 (2)	
H1N	0.6110 (15)	0.4544 (9)	0.4562 (18)	0.028 (4)*	
C1	0.85341 (10)	0.63093 (6)	0.63507 (12)	0.0172 (2)	
C2	0.84786 (10)	0.56433 (6)	0.69114 (12)	0.0165 (2)	
C3	0.82958 (10)	0.50356 (6)	0.60943 (12)	0.0155 (2)	
C4	0.81082 (10)	0.51001 (6)	0.47363 (12)	0.0159 (2)	
C5	0.81682 (10)	0.57750 (7)	0.42116 (12)	0.0181 (2)	
H5	0.8049	0.5819	0.3285	0.022*	
C6	0.83940 (10)	0.63786 (6)	0.49899 (12)	0.0188 (2)	
C7	0.85359 (10)	0.56214 (7)	0.83223 (12)	0.0193 (2)	
C8	0.82815 (14)	0.49568 (9)	1.00727 (14)	0.0332 (3)	
H8A	0.7721	0.5293	1.0178	0.050*	
H8B	0.9030	0.5076	1.0700	0.050*	
H8C	0.8077	0.4476	1.0254	0.050*	
C9	0.83582 (10)	0.42959 (6)	0.66379 (11)	0.0175 (2)	
C10	0.95930 (14)	0.34207 (8)	0.78924 (16)	0.0360 (4)	
H10A	0.9373	0.3076	0.7161	0.054*	
H10B	0.9125	0.3352	0.8460	0.054*	
H10C	1.0391	0.3352	0.8423	0.054*	
C11	0.78777 (10)	0.44813 (6)	0.38084 (11)	0.0161 (2)	
C12	0.67941 (10)	0.41792 (6)	0.32872 (11)	0.0160 (2)	
C13	0.65792 (10)	0.36524 (6)	0.23203 (12)	0.0183 (2)	
H13	0.5840	0.3457	0.1970	0.022*	
C14	0.74280 (11)	0.34054 (7)	0.18550 (12)	0.0193 (2)	
C15	0.85138 (11)	0.36831 (7)	0.24215 (13)	0.0210 (3)	
H15	0.9113	0.3506	0.2151	0.025*	
C16	0.87306 (10)	0.42142 (7)	0.33738 (12)	0.0201 (3)	
H16	0.9475	0.4399	0.3737	0.024*	
C17	0.51190 (10)	0.56517 (6)	0.25010 (12)	0.0178 (2)	
C18	0.50126 (11)	0.62124 (7)	0.32991 (13)	0.0222 (3)	
H18	0.4745	0.6133	0.4024	0.027*	
C19	0.53028 (12)	0.68894 (7)	0.30225 (13)	0.0224 (3)	
H19	0.5228	0.7273	0.3565	0.027*	
C20	0.57021 (10)	0.70204 (7)	0.19664 (12)	0.0198 (2)	
C21	0.58170 (11)	0.64480 (7)	0.11927 (12)	0.0211 (3)	
H21	0.6100	0.6526	0.0480	0.025*	
C22	0.55246 (11)	0.57649 (7)	0.14447 (12)	0.0197 (2)	
H22	0.5600	0.5380	0.0904	0.024*	
C23	0.59565 (12)	0.77675 (7)	0.16557 (13)	0.0250 (3)	
H23A	0.5250	0.8006	0.1147	0.037*	
H23B	0.6466	0.7758	0.1128	0.037*	
H23C	0.6319	0.8025	0.2489	0.037*	
C24	0.71670 (12)	0.28600 (7)	0.07589 (14)	0.0264 (3)	
H24A	0.6916	0.2420	0.1064	0.040*	
H24B	0.7849	0.2767	0.0528	0.040*	
H24C	0.6566	0.3038	−0.0027	0.040*	
C25	0.85016 (13)	0.70949 (7)	0.44329 (14)	0.0273 (3)	
H25A	0.9281	0.7264	0.4826	0.041*	
H25B	0.7985	0.7427	0.4645	0.041*	
H25C	0.8308	0.7061	0.3463	0.041*	

### Atomic displacement parameters (Å^2^)

**Table:**

	*U*^11^	*U*^22^	*U*^33^	*U*^12^	*U*^13^	*U*^23^
S1	0.01611 (15)	0.01641 (15)	0.01492 (15)	−0.00118 (10)	0.00555 (11)	−0.00276 (10)
O1	0.0258 (5)	0.0150 (4)	0.0243 (5)	−0.0007 (3)	0.0075 (4)	−0.0048 (3)
O2	0.0414 (6)	0.0258 (5)	0.0193 (4)	−0.0024 (4)	0.0098 (4)	−0.0067 (4)
O3	0.0356 (5)	0.0253 (5)	0.0150 (4)	−0.0039 (4)	0.0081 (4)	0.0016 (4)
O4	0.0268 (5)	0.0180 (4)	0.0221 (4)	−0.0035 (4)	0.0057 (4)	0.0021 (3)
O5	0.0248 (5)	0.0180 (4)	0.0243 (5)	0.0032 (4)	−0.0005 (4)	0.0032 (4)
O6	0.0185 (4)	0.0218 (4)	0.0211 (4)	−0.0015 (3)	0.0091 (4)	−0.0028 (3)
O7	0.0205 (4)	0.0210 (4)	0.0166 (4)	−0.0011 (3)	0.0041 (3)	−0.0050 (3)
N1	0.0180 (5)	0.0184 (5)	0.0138 (5)	−0.0011 (4)	0.0056 (4)	−0.0025 (4)
C1	0.0146 (5)	0.0162 (6)	0.0198 (6)	0.0002 (4)	0.0043 (4)	−0.0032 (4)
C2	0.0148 (5)	0.0173 (6)	0.0166 (5)	0.0004 (4)	0.0043 (4)	−0.0011 (4)
C3	0.0139 (5)	0.0153 (6)	0.0166 (5)	0.0002 (4)	0.0042 (4)	0.0002 (4)
C4	0.0138 (5)	0.0169 (6)	0.0163 (5)	−0.0005 (4)	0.0041 (4)	−0.0002 (4)
C5	0.0176 (5)	0.0202 (6)	0.0148 (5)	−0.0012 (4)	0.0033 (4)	0.0009 (4)
C6	0.0176 (6)	0.0165 (6)	0.0202 (6)	−0.0004 (4)	0.0036 (5)	0.0026 (5)
C7	0.0182 (6)	0.0207 (6)	0.0178 (6)	0.0016 (5)	0.0042 (5)	−0.0006 (5)
C8	0.0423 (8)	0.0419 (9)	0.0158 (6)	−0.0051 (7)	0.0099 (6)	0.0034 (6)
C9	0.0223 (6)	0.0167 (6)	0.0125 (5)	0.0014 (5)	0.0044 (4)	−0.0001 (4)
C10	0.0417 (9)	0.0202 (7)	0.0336 (8)	0.0070 (6)	−0.0043 (6)	0.0068 (6)
C11	0.0179 (6)	0.0163 (6)	0.0130 (5)	−0.0001 (4)	0.0036 (4)	0.0005 (4)
C12	0.0178 (5)	0.0152 (5)	0.0153 (5)	0.0009 (4)	0.0059 (4)	0.0014 (4)
C13	0.0186 (6)	0.0170 (6)	0.0183 (6)	−0.0010 (4)	0.0045 (5)	−0.0016 (4)
C14	0.0223 (6)	0.0170 (6)	0.0169 (5)	0.0028 (5)	0.0043 (5)	−0.0011 (4)
C15	0.0199 (6)	0.0229 (6)	0.0206 (6)	0.0042 (5)	0.0074 (5)	−0.0020 (5)
C16	0.0175 (6)	0.0226 (6)	0.0193 (6)	−0.0008 (5)	0.0048 (5)	−0.0013 (5)
C17	0.0181 (6)	0.0178 (6)	0.0163 (5)	0.0003 (4)	0.0042 (4)	−0.0008 (4)
C18	0.0284 (7)	0.0220 (6)	0.0190 (6)	−0.0007 (5)	0.0114 (5)	−0.0021 (5)
C19	0.0293 (7)	0.0184 (6)	0.0203 (6)	0.0013 (5)	0.0094 (5)	−0.0023 (5)
C20	0.0199 (6)	0.0192 (6)	0.0177 (6)	0.0006 (5)	0.0029 (5)	0.0018 (5)
C21	0.0245 (6)	0.0244 (6)	0.0150 (5)	0.0001 (5)	0.0073 (5)	0.0006 (5)
C22	0.0230 (6)	0.0206 (6)	0.0161 (5)	0.0007 (5)	0.0072 (5)	−0.0027 (5)
C23	0.0301 (7)	0.0206 (6)	0.0233 (6)	−0.0013 (5)	0.0076 (5)	0.0032 (5)
C24	0.0261 (7)	0.0250 (7)	0.0262 (7)	0.0035 (5)	0.0062 (5)	−0.0093 (5)
C25	0.0354 (7)	0.0187 (6)	0.0243 (7)	−0.0053 (5)	0.0054 (6)	0.0031 (5)

### Geometric parameters (Å, º)

**Table:**

S1—O6	1.4330 (9)	C11—C16	1.3922 (17)
S1—O7	1.4338 (9)	C11—C12	1.4056 (17)
S1—N1	1.6372 (11)	C12—C13	1.3911 (17)
S1—C17	1.7643 (13)	C13—C14	1.3940 (17)
O1—C1	1.3543 (14)	C13—H13	0.9500
O1—H1O	0.91 (2)	C14—C15	1.3942 (18)
O2—C7	1.2239 (16)	C14—C24	1.5076 (17)
O3—C7	1.3203 (16)	C15—C16	1.3864 (18)
O3—C8	1.4507 (16)	C15—H15	0.9500
O4—C9	1.2020 (16)	C16—H16	0.9500
O5—C9	1.3425 (15)	C17—C18	1.3907 (17)
O5—C10	1.4444 (16)	C17—C22	1.3952 (17)
N1—C12	1.4298 (15)	C18—C19	1.3864 (18)
N1—H1N	0.836 (18)	C18—H18	0.9500
C1—C2	1.4029 (17)	C19—C20	1.3955 (18)
C1—C6	1.4054 (17)	C19—H19	0.9500
C2—C3	1.4112 (16)	C20—C21	1.3943 (18)
C2—C7	1.4794 (17)	C20—C23	1.5064 (17)
C3—C4	1.3899 (16)	C21—C22	1.3904 (18)
C3—C9	1.5031 (16)	C21—H21	0.9500
C4—C5	1.4028 (17)	C22—H22	0.9500
C4—C11	1.4941 (16)	C23—H23A	0.9800
C5—C6	1.3811 (17)	C23—H23B	0.9800
C5—H5	0.9500	C23—H23C	0.9800
C6—C25	1.4999 (17)	C24—H24A	0.9800
C8—H8A	0.9800	C24—H24B	0.9800
C8—H8B	0.9800	C24—H24C	0.9800
C8—H8C	0.9800	C25—H25A	0.9800
C10—H10A	0.9800	C25—H25B	0.9800
C10—H10B	0.9800	C25—H25C	0.9800
C10—H10C	0.9800		
			
O6—S1—O7	119.80 (5)	C13—C12—C11	120.39 (11)
O6—S1—N1	104.85 (5)	C13—C12—N1	119.43 (11)
O7—S1—N1	107.72 (5)	C11—C12—N1	120.16 (10)
O6—S1—C17	108.52 (6)	C12—C13—C14	121.23 (11)
O7—S1—C17	107.68 (6)	C12—C13—H13	119.4
N1—S1—C17	107.72 (6)	C14—C13—H13	119.4
C1—O1—H1O	104.7 (14)	C13—C14—C15	118.09 (11)
C7—O3—C8	116.17 (11)	C13—C14—C24	120.53 (11)
C9—O5—C10	115.05 (11)	C15—C14—C24	121.37 (11)
C12—N1—S1	123.00 (8)	C16—C15—C14	120.91 (11)
C12—N1—H1N	116.8 (12)	C16—C15—H15	119.5
S1—N1—H1N	111.3 (12)	C14—C15—H15	119.5
O1—C1—C2	122.66 (11)	C15—C16—C11	121.26 (11)
O1—C1—C6	116.37 (11)	C15—C16—H16	119.4
C2—C1—C6	120.97 (11)	C11—C16—H16	119.4
C1—C2—C3	119.15 (11)	C18—C17—C22	120.65 (12)
C1—C2—C7	117.65 (11)	C18—C17—S1	119.52 (10)
C3—C2—C7	123.06 (11)	C22—C17—S1	119.82 (9)
C4—C3—C2	120.39 (11)	C19—C18—C17	119.11 (12)
C4—C3—C9	116.77 (10)	C19—C18—H18	120.4
C2—C3—C9	122.74 (10)	C17—C18—H18	120.4
C3—C4—C5	118.70 (11)	C18—C19—C20	121.58 (12)
C3—C4—C11	123.18 (11)	C18—C19—H19	119.2
C5—C4—C11	118.10 (10)	C20—C19—H19	119.2
C6—C5—C4	122.53 (11)	C21—C20—C19	118.23 (12)
C6—C5—H5	118.7	C21—C20—C23	121.65 (12)
C4—C5—H5	118.7	C19—C20—C23	120.08 (12)
C5—C6—C1	118.13 (11)	C22—C21—C20	121.27 (11)
C5—C6—C25	122.27 (11)	C22—C21—H21	119.4
C1—C6—C25	119.60 (11)	C20—C21—H21	119.4
O2—C7—O3	122.41 (12)	C21—C22—C17	119.15 (11)
O2—C7—C2	123.45 (12)	C21—C22—H22	120.4
O3—C7—C2	114.12 (11)	C17—C22—H22	120.4
O3—C8—H8A	109.5	C20—C23—H23A	109.5
O3—C8—H8B	109.5	C20—C23—H23B	109.5
H8A—C8—H8B	109.5	H23A—C23—H23B	109.5
O3—C8—H8C	109.5	C20—C23—H23C	109.5
H8A—C8—H8C	109.5	H23A—C23—H23C	109.5
H8B—C8—H8C	109.5	H23B—C23—H23C	109.5
O4—C9—O5	124.64 (11)	C14—C24—H24A	109.5
O4—C9—C3	124.75 (11)	C14—C24—H24B	109.5
O5—C9—C3	110.41 (10)	H24A—C24—H24B	109.5
O5—C10—H10A	109.5	C14—C24—H24C	109.5
O5—C10—H10B	109.5	H24A—C24—H24C	109.5
H10A—C10—H10B	109.5	H24B—C24—H24C	109.5
O5—C10—H10C	109.5	C6—C25—H25A	109.5
H10A—C10—H10C	109.5	C6—C25—H25B	109.5
H10B—C10—H10C	109.5	H25A—C25—H25B	109.5
C16—C11—C12	118.01 (11)	C6—C25—H25C	109.5
C16—C11—C4	120.09 (11)	H25A—C25—H25C	109.5
C12—C11—C4	121.80 (11)	H25B—C25—H25C	109.5
			
O6—S1—N1—C12	173.68 (9)	C3—C4—C11—C16	−102.44 (14)
O7—S1—N1—C12	45.03 (11)	C5—C4—C11—C16	75.74 (15)
C17—S1—N1—C12	−70.86 (11)	C3—C4—C11—C12	81.40 (15)
O1—C1—C2—C3	−178.41 (11)	C5—C4—C11—C12	−100.42 (14)
C6—C1—C2—C3	0.95 (17)	C16—C11—C12—C13	−2.95 (17)
O1—C1—C2—C7	5.80 (17)	C4—C11—C12—C13	173.28 (11)
C6—C1—C2—C7	−174.84 (11)	C16—C11—C12—N1	175.10 (11)
C1—C2—C3—C4	−3.68 (17)	C4—C11—C12—N1	−8.66 (17)
C7—C2—C3—C4	171.87 (11)	S1—N1—C12—C13	−65.05 (14)
C1—C2—C3—C9	172.71 (11)	S1—N1—C12—C11	116.88 (11)
C7—C2—C3—C9	−11.74 (18)	C11—C12—C13—C14	0.72 (18)
C2—C3—C4—C5	3.56 (17)	N1—C12—C13—C14	−177.35 (11)
C9—C3—C4—C5	−173.04 (10)	C12—C13—C14—C15	2.31 (19)
C2—C3—C4—C11	−178.27 (11)	C12—C13—C14—C24	−176.98 (12)
C9—C3—C4—C11	5.13 (17)	C13—C14—C15—C16	−3.09 (19)
C3—C4—C5—C6	−0.73 (18)	C24—C14—C15—C16	176.20 (12)
C11—C4—C5—C6	−178.99 (11)	C14—C15—C16—C11	0.8 (2)
C4—C5—C6—C1	−1.93 (18)	C12—C11—C16—C15	2.20 (18)
C4—C5—C6—C25	177.57 (12)	C4—C11—C16—C15	−174.11 (11)
O1—C1—C6—C5	−178.81 (11)	O6—S1—C17—C18	18.25 (12)
C2—C1—C6—C5	1.80 (18)	O7—S1—C17—C18	149.32 (10)
O1—C1—C6—C25	1.68 (17)	N1—S1—C17—C18	−94.76 (11)
C2—C1—C6—C25	−177.72 (12)	O6—S1—C17—C22	−162.58 (10)
C8—O3—C7—O2	2.03 (19)	O7—S1—C17—C22	−31.51 (12)
C8—O3—C7—C2	−176.26 (11)	N1—S1—C17—C22	84.42 (11)
C1—C2—C7—O2	−8.10 (18)	C22—C17—C18—C19	0.8 (2)
C3—C2—C7—O2	176.29 (12)	S1—C17—C18—C19	179.92 (10)
C1—C2—C7—O3	170.16 (11)	C17—C18—C19—C20	−0.2 (2)
C3—C2—C7—O3	−5.45 (17)	C18—C19—C20—C21	−0.7 (2)
C10—O5—C9—O4	−3.35 (18)	C18—C19—C20—C23	177.09 (12)
C10—O5—C9—C3	−178.35 (11)	C19—C20—C21—C22	1.12 (19)
C4—C3—C9—O4	−68.07 (16)	C23—C20—C21—C22	−176.63 (12)
C2—C3—C9—O4	115.42 (14)	C20—C21—C22—C17	−0.61 (19)
C4—C3—C9—O5	106.91 (12)	C18—C17—C22—C21	−0.35 (19)
C2—C3—C9—O5	−69.60 (14)	S1—C17—C22—C21	−179.51 (9)

### Hydrogen-bond geometry (Å, º)

*Cg*1 is the centroid of the C1–C6 ring.

**Table:**

*D*—H···*A*	*D*—H	H···*A*	*D*···*A*	*D*—H···*A*
N1—H1*N*···O4	0.836 (18)	2.507 (18)	3.0185 (14)	120.5 (15)
N1—H1*N*···O6^i^	0.836 (18)	2.280 (18)	3.0643 (14)	156.4 (18)
O1—H1*O*···O2	0.91 (2)	1.72 (2)	2.5596 (14)	152 (2)
C13—H13···O7	0.95	2.53	3.0022 (16)	111
C18—H18···O6	0.95	2.58	2.9370 (16)	103
C24—H24*A*···O4^ii^	0.98	2.60	3.3784 (16)	137
C25—H25*C*···O1^iii^	0.98	2.59	3.2177 (17)	122
C16—H16···*Cg*1^iv^	0.95	2.61	3.4780 (14)	153

Symmetry codes: (i) −*x*+1, −*y*+1, −*z*+1; (ii) *x*, −*y*+1/2, *z*−1/2; (iii) *x*, −*y*+3/2, *z*−1/2; (iv) −*x*+2, −*y*+1, −*z*+1.
